# Papanicolaou smears and cervical inflammatory cytokine responses

**DOI:** 10.1186/1476-9255-4-8

**Published:** 2007-04-24

**Authors:** Jo-Ann S Passmore, Chelsea Morroni, Samual Shapiro, Anna-Lise Williamson, Margaret Hoffman

**Affiliations:** 1 Division of Medical Virology, Institute of Infectious Disease and Molecular Medicine, Faculty of Health Sciences, University of Cape Town, Cape Town, South Africa; 2 Women's Health Research Unit, School of Public Health and Family Medicine, Faculty of Health Sciences, University of Cape Town, Cape Town, South Africa; 3 Department Of Epidemiology, Mailman School of Public Health, New York, USA; 4 National Health Laboratory Service, Groote Schuur Hospital, Observatory, Cape Town, South Africa

## Abstract

In a case-control study among 2064 South African women to investigate the risk of clinically invasive cancer of the cervix, we found a marked reduction in the risk of cervical cancer among women who gave a history of ever having undergone even a single Pap smear, and a statistically significant decline in the HPV positivity rate correlated with the lifetime number of Pap smears received. HPV infections and their associated low-grade lesions commonly regress, indicating that most often there is an effective host immune response against HPV infection. We hypothesized that act of performing a Pap smear is associated with inflammatory responses at the site of trauma, the cervix, and that this inflammatory signalling may be an immunological factor initiating these productive anti-HPV responses. In the present study, a randomized controlled trial, we enrolled 80 healthy young women to investigate the impact of performing a Pap smear on cervical inflammation. Forty one women, in the intervention group, received a Pap smear at enrollment and cervicovaginal lavages (CVLs) were collected at baseline and 2 weeks later. Thirty nine women received no intervention at enrollment (control group) but CVLs were collected at enrolment and 2 weeks later. We assessed various markers of inflammation including IL-12 p70, TNF-α, IL-8, IL-6, IL-10, and IL-1β in CVL specimens. While CVL levels of IL-8, IL-1β and IL-6 remained unchanged following a Pap smear, markers of cell mediated immunity (IL-12 p70 and TNF-α) and T cell regulation (IL-10) were significantly elevated.

## Background

In South Africa and worldwide, cervical cancer is the second most common cancer in women with an overall age standardized incidence rate of 30 per 100,000 [1]. Cervical cancer is predominantly a sexually transmitted disease associated with infection with certain types of the human papillomavirus (HPV) [2]. Internationally it has been shown that screening for precursors of cervical cancer, most commonly by means of Papanicoloau (Pap) smears, substantially reduces the incidence of invasive cancer [3-6]. We have recently completed a case-control study among 2064 South African women to investigate the risk of clinically invasive cancer of the cervix in relation to hormonal contraceptives use [7]. We found both a marked reduction in the risk of cervical cancer among women who gave a history of ever having undergone even a single Pap smear, and a 50% reduction in HPV prevalence among woman who had undergone two or more smears. There was a statistically significant decline in the HPV positivity rates according to the lifetime number of Pap smears women had received [8].

HPV infections and their associated low-grade lesions commonly regress [9], indicating that there is most often an effective host immune response against HPV infection. Regression of anogenital warts is associated with infiltration of T cells [10] and it is generally thought that regression is largely driven by HPV-specific immunity. We hypothesize here that the minor trauma and associated inflammatory responses involved in taking a Pap smear may be an important factor initiating these productive responses. This hypothesis is supported by the clinical observation that genital condylomas and warts, also known to be caused by specific HPV types [11], usually regress, often without recurrence, following cauterization. Furthermore, the effectiveness of topically applied Imiquimod, an immune response modifier, in the treatment of patients with HPV-associated genital and peri-anal warts is well documented and has largely been ascribed to initiation of inflammatory cytokine response cascades [12].

The aim of this study was to investigate the impact of minor trauma to the cervix caused by a Pap smear on local mucosal inflammatory responses. Comparing cervicovaginal lavage (CVL) specimens from healthy young women who had recently received a Pap smear with those that had not, we assessed various markers of inflammation including (i) interleukin (IL)-12 p70 and tumour necrosis factor (TNF)-α (associated with Th1 protective responses); (ii) IL-8 (associated with neutrophil recruitment); (iii) IL-6 (associated with B cell recruitment); IL-10 (associated with T cell regulation) and IL-1β (associated with leukocyte recruitment, activation of NFκB and upstream induction of other cytokines, prostanoids and nitric oxide associated with inflammation) [13-15].

## Methods

A randomised controlled trial was conducted in which **w**omen between the ages of 18 and 29 years were recruited from the University of Cape Town Student Health Clinic, Cape Town, South Africa. Women were eligible to participate if they had been resident in the study area for at least 6 months and had no previous history of malignancy at any site. Women were ineligible if they had (i) a current sexually transmitted infection (reported or on examination), (ii) undergone a Pap smear within the previous 6 months, or (iii) used a vaginal medication during the week prior to Visit 1 (baseline). Eligible and consenting women were randomly assigned to either the intervention or the control group. Women in the intervention group received a cervico-vaginal lavage (CVL) followed immediately by a Pap smear (at baseline), while women in the control group received CVL at baseline, but only received a Pap smear upon exit from the study (at Visit 2, after 2 weeks of follow-up from Visit 1). To normalize for potential changes in cytokine and/or protein concentrations during the menstrual cycle [16,17] women were enrolled 2–3 days following the last day of menses (baseline during which the intervention group received a Pap smear and both groups received CVL). Both intervention and control participants were asked to return to the clinic 14 days later for the follow up visit (during which both groups received a second CVL). Following this second CVL, the women in the control group received a Pap smear.

Information on socio-demographic characteristics, reproductive history and sexual activity were collected from participants by a questionnaire administered by the nurse interviewer. The Research Ethics Committee of the Faculty of Health Sciences, University of Cape Town approved the study and written informed consent was obtained from all women before participation.

### Pap smears

Pap smears were taken using the Aylesbury spatula by placing it in the cervical os and rotating it through 360°. This instrument was used for taking Pap smears as it is the one employed in the public health sector clinics in South Africa. A cytologist screened the slides. Women with abnormal Pap smears were referred to appropriate services for management.

### Collection and processing of cervical specimens

After consent, a vaginal examination was performed on all participants. In the intervention group at baseline, the CVL was performed immediately prior to the Pap smear. In the control group, only a CVL was performed at baseline. The CVL was performed by inserting 6 ml sterile PBS into the external cervical os and irrigating the endocervical region for approximately 30 seconds using a sterile disposable plastic Pasteur pipette. The CVL fluid pooled in the posterior fornix of the vagina was then withdrawn using the same pipette. Good recovery of 5.5 – 6.0 ml PBS per patient was recorded. The fluid was then transferred into a sterile transport tube and transported to the laboratory at 4°C within 2 hours of collection. CVLs were processed immediately in the laboratory by centrifugation at 1000 × g for 10 minutes at 4°C and the aliquoted supernatants stored at -80°C until further processing.

### Determination of CVL protein concentrations

Protein concentrations in paired patient CVLs (baseline and follow-up) were evaluated using the commercial BCA Protein Assay Kit (Pierce, Rockford, IL, USA) according the manufacturer's instructions.

### Determination of inflammatory cytokine profiles in CVLs

Cervical cytokine responses were evaluated directly *ex vivo *by evaluating inflammatory cytokine production profiles in the supernatant fraction of the CVL sample using the commercial Becton Dickenson Human Inflammation Cytometric Bead Array (CBA) system and FACS analysis, according the manufacturer's instructions. This system allowed for detection of IL-8, IL-1β, IL-6, IL-10, TNF-α and IL-12p70 per single patient specimen. Inflammatory cytokine concentrations in the supernatant fraction of the CVL sample were analyzed according the manufacturer's instructions (BD Biosciences, San Diego, CA, USA). Fifty μl of CVL was used per participant and each participant sample was analyzed once. The sensitivity of this system was between 1.9 and 7.2 pg/mL for each of the six cytokines. Samples with cytokine levels below the lower limit of detection of the assay were reported as zero, and those above the upper detection limit of 5000 pg/mL were assigned a value of 5000 pg/mL.

### Statistical analysis

Statistical analyses were performed using Statistica^® ^and GraphPad Prism^®^. Unless otherwise indicated, the Mann-Whitney U Test was applied for independent sample comparisons, the Wilcoxon Ranks Test was applied for matched-pair comparisons and Spearman Ranks for correlations. For comparison of cervical protein concentrations, the Student's t-test for independent and dependent samples was applied as indicated. *P*-values ≤ 0.05 were considered statistically significant.

## Results

### Description of participants and randomization

Of the 90 women recruited, 9 were not eligible to participate for the following reasons: reported recent/current genital herpes (n = 4), pregnant in the past 6 months (n = 2), inter-uterine device in the past 6 months (n = 1) and never had vaginal sexual intercourse (n = 2). The remaining 80 eligible women all consented to participate. Participants were randomly assigned to either the intervention (n = 41) or the control group (n = 40). No participants withdrew from the study, but one in the control group was lost to follow-up (intervention group = 41; control = 39).

Table 1 summarizes the socio-demographic, reproductive and sexual characteristics by randomization group; no significant differences were observed between these groups at baseline or at follow-up (in terms of any vaginal intercourse, number of acts of vaginal intercourse, condom use and use of any vaginal medications during the study follow-up period, data not shown). No participants received a Pap smear outside the study during the study period. There were 4/39 women in the control group and 4/41 women in the intervention group (Pap smears) who had abnormal Pap smears during the study period (p = 0.95; *Χ*^2 ^test). In the control group, 3/4 women with abnormal Pap smears had LSIL while 1/4 had ASCUS. In the intervention group, 2/4 women with abnormal Pap smears had LSIL while 2/4 had ASCUS.

**Table 1:** Socio-demographic, reproductive and sexual characteristics by randomization group at enrolment

Characteristic	Intervention: received a Pap Smear (n = 41)	Control: did not receive a Pap Smear (n = 39)	*P*-value
Age [median years (IQR)]	21.5 (20.0–23.0)	21 (20.0–22.8)	0.4
Vaginal intercourse during follow-up [n (%)]	21 (51.2)	17 (43.6)	0.5
Number of acts of vaginal intercourse [median years (IQR)]	3 (1.5–4.5)	1 (1–2)	0.1
Abnormal Pap smear during study [n (%)]	4 (9.8)	4 (10.3)	0.9
Ever pregnant [n (%)]	3 (7.1)	8 (20.0)	0.1
Number of live births among ever pregnant [n (%)]	0 (0.0)	2 (25.0)	0.2
Ever Pap smear [n (%)]	13 (30.9)	8 (20.0)	0.3
Number of Pap smears [median (IQR)]	2 (1–2)	1 (1–1.8)	0.2
Currently using hormonal contraception [n (%)]	19 (45.2)	17 (42.5)	0.8
Currently using [n(%)]			
Condom	28 (66.7)	32 (80.0)	0.2
Combined oral contraceptive pills	13 (30.9)	8 (20.0)	0.3
Injectable	6 (14.6)	9 (22.5)	0.3
Ever vaginal discharge of concern [n (%)]	14 (33.3)	19 (47.5)	0.2
Age first vaginal intercourse [median years (IQR)]	18 (17–19)	17 (17–18.8)	0.4
Lifetime no. of sexual partners [median (IQR)]	2 (1–4)	2 (2–3)	0.5
Ever condom use [n (%)]	39 (92.8)	38 (95.0)	0.7
Always condom use [n (%)]	17 (43.5)	18 (47.4)	0.7
Rarely condom use [n (%)]	5 (12.8)	5 (13.2)	0.9
Use tampons when menstruating [n (%)]	25 (59.9)	26 (65.0)	0.6

### Quantification of total protein concentrations in CVLs

**Figure 1:**
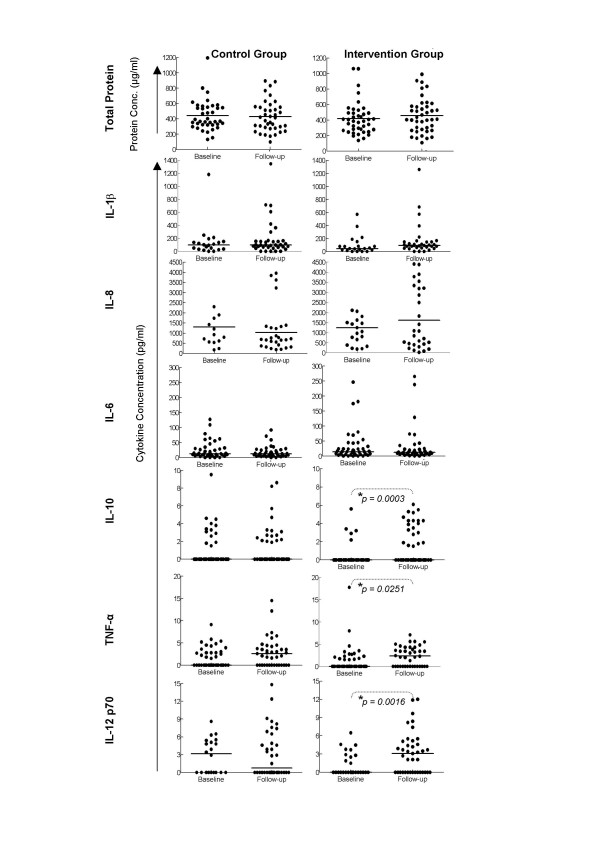
**Inflammatory cytokine and protein concentrations in cervicovaginal lavages (CVL) from women having received a Pap smear (intervention group; right panel) compared to women who had not (control group; left panel)**. Total protein (A), IL-1β (B), IL-8 (C), IL-6 (D), IL-10 (E), TNF-α (F) and IL-12 p70 (G) concentrations were measured in the CVL fluid from each woman using the BD CBA Inflammation panel and FACS analysis. Each (●) represents an individual woman's protein or cytokine concentration. Solid lines indicate the median concentration for each group. P-values were calculated using Wilcoxon Ranks test for matched non-parametric data and p-values ≤ 0.05 were considered significant.

Total protein in CVLs was determined at baseline (+3 days post-menses) and at follow-up (14 days later or +17 days post-menses) (Figure 1A). No significant difference in total protein concentrations in CVLs was observed within or between randomization groups.

### Effect of Pap smear on cervical concentrations of inflammatory cytokines

CVLs from each participant were investigated for the presence of cervical inflammatory cytokines (IL-8, IL-1β, IL-6, TNF-α, IL-12 p70 and IL-10) at baseline and follow-up (Figure 1; see also Additional file 1 and 2). We found that levels of IL-1β (Figure 1B), IL-8 (Figure 1C), and IL-6 (Figure 1D) did not differ significantly between baseline and follow-up in either the control group (left panels) or the intervention group (right panels) indicating that a Pap smear was not detectably associated with increases in these proinflammatory cytokines. In contrast, women who received a Pap smear (intervention group) showed significantly increased levels of the Th1 associated cytokines TNF-α (Figure 1F; p = 0.025) and IL-12 p70 (Figure 1G; p = 0.0016) and the regulatory cytokine IL-10 (Figure 1E; p = 0.0003). When these cytokine responses were scored according to whether the women had a detectable cytokine response or not, significantly more women from the intervention group had increased levels of IL-12 p70 (p = 0.0007; *Χ*^2 ^test) and IL-10 (p = 0.0003; *Χ*^2 ^test) (Table 2).

**Table 2:** Comparison of the number of women who received a Pap smear compared to controls who had detectable levels of inflammatory cytokines.^a^

Cytokine	N	Intervention (Number of Responders^a^; %)		*P-value*	N	Control (Number of Responders^a^; %)		*P-value*
						
		Baseline	Follow-up			Baseline	Follow-up	
IL-12 p70	34	10/34 (29.4)	24/34 (70.6)	*0.0007*	36	11/36 (30.6)	18/36 (50.0)	0.0926
TNF-α	41	16/41 (39.0)	24/41 (58.5)	0.0772	39	19/39 (48.7)	25/39 (64.1)	0.1707

IL-10	41	5/41 (12.2)	20/41 (48.8)	*0.0003*	39	13/39 (33.3)	16/39 (41.0)	0.4821

^a^Responders are defined as participants with cytokine levels above the detectable limit of the assay.

## Discussion

In this study, we investigated the impact of minor trauma to the cervix caused by a Pap smear on local mucosal inflammatory responses. Concentrations of the inflammatory cytokines IL-12 and TNF-α and the regulatory cytokine IL-10 were significantly increased in the genital secretions (cervicovaginal lavage) of women who received Pap smears compared with women who received no intervention. In addition, significantly more women from the intervention group had increased levels of IL-12 p70 and IL-10. This is the first study to our knowledge to investigate the effect of a Pap smear on cervical inflammation. It remains to be determined whether this mucosal inflammatory response is linked to a lower incidence of HPV infection.

There have been studies demonstrating that repetitive sexual intercourse was associated with long-term protection from HPV infection and the proposed mechanism for this was inflammation associated with repeated insult [18]. Studies on the topical immune response modifier, Imiquimod [12,19,20], used in the treatment genital and peri-anal warts provide further evidence that mild inflammation may be protective against HPV infection. Imiquimod has been shown to activate both innate and cell mediated immunity with local induction of inflammatory cytokines IFN-α, IFN-γ, and TNF-α, [21-23]. Imiquimod-mediated wart reduction was also associated with significant decreases in HPV DNA copy number/cell [12,23] and these studies have confirmed that this is largely due to local inflammatory mediators.

The levels of IL-12 p70, IL-10 and TNF-α measured in this study were quite low raising some concern about the biological significance of the changes observed between groups. Despite this, concentrations of cytokines measured in this study were comparable to levels published elsewhere when CVL [24] and cervical mucous were assessed [25] confirming that these cytokines are present and active at low concentrations. Evidence from vaginal microbicide studies have shown that even low concentrations of IL-1β and IL-6 (as little as a 9 pg/ml induction of IL-1β) correlate significantly with vaginal irritation and inflammation following application of vaginal microbicides implying associated biological activity [26]. Finally, the levels of TNF-α following cryo- or loop surgical treatment of the cervix were shown to increase to a maximum of 60 pg/ml at 14 days and this was associated with peak inflammation and significant macroscopic ulceration of the cervix [27]. Based on these modest levels following more severe inflammatory interventions (surgery) than used in this study (Pap smear), we argue that the small changes observed following a Pap smear are biologically relevant.

Since little data is available on the impact of female sex hormones on cervical inflammatory cytokine responses, our study normalized for menstral cycle changes by obtaining CVLs from women 3 days post menses and 10 days later. Interestingly, we did not observe any significant changes in the inflammatory cytokines assessed in the control arm of the study. Although there is sound evidence that levels of cervicovaginal antibodies are linked to phases of the menstral cycle and that sex hormones impact on this, there is no similar consensus on these effects on mucosal cytokine secretion [28]. There have been reports that levels of IL-10 and IL-1β are elevated at the time of ovulation whereas IL-6 cervical concentrations do not correlate with the ovulatory cycle or female sex hormone levels [25]. In contrast, White et al. [28] showed that intraepithelial lymphocyte and CTL activity persisted in the vagina and cervix throughout the menstrual cycle but was absent in the uterus during the secretory phase of the cycle. Since inflammatory cytokines are secreted by both epithelial cells and intraepithelial lymphocytes, it is possible that sex hormone regulation of epithelial cells may impact epithelial cell cytokine production [29,30]. We found no evidence to support this.

In conclusion, this study provides the first evidence that a Pap smear does significantly up-regulate levels of inflammatory cytokines IL-12, TNF-α and IL-10 at the cervix. This is an important step towards understanding whether these local Pap smear-associated inflammatory responses are one of the factors initiating more long-term protection from HPV infection and clearance.

## Supplementary Material

Additional File 1 Mean and SD of inflammatory cytokine levels in women who received a Pap smear compared to controls. The data provided represents the statistical analysis of mean and standard deviation of respective inflammatory cytokine levels in women who received a Pap smear compared to women who did not. Click here for file

Additional File 2 Median and IQR of inflammatory cytokine levels in women who received a Pap smear compared to controls. The data provided represents the statistical analysis of median and interquartile ranges of respective inflammatory cytokine levels in women who received a Pap smear compared to women who did not. Click here for file
