# Supplementary material for: Papanicolaou smears and cervical inflammatory cytokine responses
Source: J Inflamm (Lond). 2007 Apr 24;4:8. doi: 10.1186/1476-9255-4-8 (PMC1868022; doi:10.1186/1476-9255-4-8)
Supplement: Additional File 1 — Mean and SD of inflammatory cytokine levels in women who received a Pap smear compared to controls. The data provided represents the statistical analysis of mean and standard deviation of respective inflammatory cytokine levels in women who received a Pap smear compared to women who did not. [file 1476-9255-4-8-S1.doc]

Additional Table 1. Mean and SD of inflammatory cytokine levels in women who received a Pap smear compared to controls.

| Cytokine | N | Intervention (pg/ml) | | Difference (pg/ml) | *P-value^a^* | N | Control (pg/ml) | | Difference (pg/ml) | *P-value^a^* | Diff_Int_ - Diff_Con_ |
| --- | --- | --- | --- | --- | --- | --- | --- | --- | --- | --- | --- |
|  |  | Baseline | Follow-up |  |  |  | Baseline | Follow-up |  |  |  |
| IL-12 p70  TNF-α | 34  41 | 0.83 (± 1.72)  1.51 (± 3.14) | 3.49 (± 3.57)  3.99 (± 10.76) | 2.66  3.48 | *0.0016*  *0.0251* | 36  39 | 1.59 (± 2.57)  1.81 (± 2.26) | 3.17 (± 4.02)  2.88 (± 3.30) | 0.31  1.07 | *0.27*  *0.65* | 2.35  2.41 |
| IL-10 | 41 | 0.42 (± 1.22) | 1.79 (± 2.09) | 1.37 | *0.0003* | 39 | 2.03 (± 5.38) | 2.09 (± 4.23) | 0.06 | *0.68* | 1.31 |
| IL-1β  IL-8  IL-6 | 34  34  41 | 99.98 (± 141.73)  1621.03 (± 1554.76)  33.41 (± 52.68) | 163.52 (± 242.13)  2230.7 (± 1881.8)  30.66 (± 56.16) | 63.54  609.7  0.0 | *0.12*  *0.69*  *0.73* | 36  36  39 | 148.29 (± 253.57)  2401.27 (± 2025.13)  25.7 (± 29.75) | 189.86 (± 270.52)  2090.67 (± 1966.75)  18.38 (± 19.37) | 41.57  0.0  0.0 | *0.48*  *0.97*  *0.16* | 21.97  609.7  0.0 |

^a^Wilcoxon Rank Test for paired non-parametric data
