# Supplementary material for: Papanicolaou smears and cervical inflammatory cytokine responses
Source: J Inflamm (Lond). 2007 Apr 24;4:8. doi: 10.1186/1476-9255-4-8 (PMC1868022; doi:10.1186/1476-9255-4-8)
Supplement: Additional File 2 — Median and IQR of inflammatory cytokine levels in women who received a Pap smear compared to controls. The data provided represents the statistical analysis of median and interquartile ranges of respective inflammatory cytokine levels in women who received a Pap smear compared to women who did not. [file 1476-9255-4-8-S2.doc]

Additional Table 2. Median and IQR of inflammatory cytokine levels in women who received a Pap smear compared to controls.

| Cytokine | N | Intervention (pg/ml) | | *P-value^a^* | N | Control (pg/ml) | | *P-value^a^* |
| --- | --- | --- | --- | --- | --- | --- | --- | --- |
|  |  | Baseline | Follow-up |  |  | Baseline | Follow-up |  |
| IL-12 p70  TNF-α | 34  41 | 0.0 (0.0 – 0.0)  0.0 (0.0 – 2.2) | 3.5 (0.0 – 5.3)  2.4 (0.0 – 4.3) | *0.0016*  *0.0251* | 36  39 | 0.0 (0.0 – 3.5)  0.0 (0.0 – 2.9) | 0.8 (0.0 – 5.3)  2.6 (0.0 – 4.0) | *0.27*  *0.65* |
| IL-10 | 41 | 0.0 (0.0 – 0.0) | 0.0 (0.0 – 3.9) | *0.0003* | 39 | 0.0 (0.0 – 2.8) | 0.0 (0.0 – 2.7) | *0.68* |
| IL-1β  IL-8  IL-6 | 34  34  41 | 44.4 (25.6 – 79.4)  1248.5 (662.6 – 1805.2)  14.7 (5.5 – 32.3) | 95.0 (62.6 – 145.6)  1618.2 (476.5 – 3859.5)  12.9 (7.0 – 22.4) | *0.12*  *0.69*  *0.73* | 36  36  39 | 96.2 (33.8 – 141.3)  1569.9 (681.8 – 5000.0)  12.5 (5.2 – 25.0) | 96.2 (67.5 – 147.5)  1038.7 (578.1 – 4208.8)  12.7 (4.8 – 17.3) | *0.48*  *0.97*  *0.16* |

^a^Wilcoxon Rank Test for paired non-parametric data
